# PKC in the perspective of dopamine receptor signaling

**DOI:** 10.3389/abp.2025.14488

**Published:** 2025-06-09

**Authors:** Haixiang Ma

**Affiliations:** School of Pharmaceutical Sciences, Guizhou University, Guiyang, China

**Keywords:** protein kinase C, dopamine receptor, signal transduction, physiological function, dopaminergic disorders

## Abstract

Protein kinase C (PKC) is widely distributed in various tissues, organs, and cells. By catalyzing the phosphorylation of Ser/Thr residues on various proteins, PKC regulates the metabolism, growth, proliferation, and differentiation of multiple cells and plays a crucial role in transmembrane signal transmission. In dopamine receptor signal transduction, PKC regulates numerous physiological functions, such as dopamine release, internalization of the dopamine transporter, downregulation of dopamine receptors, etc. In disease conditions, hyperactivation of PKC can lead to disorders such as schizophrenia and Parkinson’s disease, while reduced PKC signaling may be associated with Alzheimer’s disease. In the past few decades, researchers have paid increasing attention to the transduction role of PKC in dopamine receptor signaling, aiming to identify and discover potential targets for dopaminergic diseases. This review, from the perspective of signal transduction between dopamine receptors and PKC, reveals the pivotal hub position of PKC in the intracellular signal transduction network and its regulation of various physiological functions, providing ideas for future research on PKC and therapeutic interventions for dopaminergic diseases.

## Introduction

The protein kinase C (PKC) family, like protein kinase A (PKA), is a class of serine/threonine kinases. It consists of a single peptide chain with a molecular weight ranging from 67 to 83 kDa. Currently, much evidence has confirmed that there are at least 11 subtypes of PKC, which are widely distributed in various tissues, organs, and cells. Different PKC subtypes have different requirements for second messengers and functions. Therefore, they can be subdivided into three subfamilies: conventional PKC (cPKC), novel PKC (nPKC), and atypical PKC (aPKC). Members of the conventional PKC (cPKC) family include PKC-α, PKC-βI, PKC-βII, and PKC-γ. They rely on the coordinated activation of calcium ions and diacylglycerol (DAG) and participate in important functions such as cell proliferation and differentiation. Members of the novel PKC (nPKC) family include PKC-δ, PKC-ε, PKC-η, and PKC-θ. Unlike cPKC, nPKC is not dependent on calcium ions but is directly activated by DAG. nPKC is mainly involved in the regulation of inflammatory responses and cell survival. Atypical PKC (aPKC) includes PKC-ζ and PKC-ι/λ. aPKC is not dependent on calcium ions or DAG but is regulated by other signal molecules, such as phosphatidic acid (PA), and is mainly closely related to cell polarity, metabolism, and embryonic development (Freeley et al., 2011; Mellor and Parker, 1998; Neve et al., 2004; Newton, 2001; Sajan et al., 2021). Due to the wide distribution of PKC, once activated, it conducts signal transduction to its downstream substrates. Molecules such as extracellular regulated protein kinases (ERK) and IκB carry out corresponding physiological activities. In different cells, PKC participates in different physiological activities. For example, in smooth muscle cells, PKC participates in cell contraction and relaxation (Kim et al., 2013); in cancer cells, PKC participates in cell proliferation and invasion (He et al., 2022; Su et al., 2013); in mammalian oocytes, PKC participates in the fertilization process of oocytes (Halet, 2004); in immune cells, PKC participates in immune regulation (Gruber et al., 2009); in nerve cells, PKC participates in the regulation of mental diseases (Saito and Shirai, 2002).

Dopamine receptors (DR) are members of the G protein-coupled receptor (GPCR) family and are a class of neurotransmitter receptors that regulate motor control, cognition, emotion, incentive mechanisms, reward, and endocrine regulation. There are currently five known subtypes of dopamine receptors (D_1_R, D_2_R, D_3_R, D_4_R, and D_5_R) (Channer et al., 2023; Kawahata and Fukunaga, 2023). According to the different G protein subunits they couple to, they are divided into two major categories: D1-like receptors and D2-like receptors. D1-like receptors are composed of D_1_R and D_5_R. D1-like receptors couple to the Gαs protein. Through the Gαs protein, they promote the activity of adenylate cyclase (AC), increase the intracellular cAMP level, and thus activate pathways such as PKA, ultimately causing various intracellular effects, such as gene expression regulation and neural signal transmission. They are mainly distributed in regions such as the striatum, frontal lobe, and limbic system (Mishra et al., 2018; Yang, 2021). D2-like receptors are composed of D_2_R, D_3_R, and D_4_R. D2-like receptors couple to the Gαi protein. Unlike D1-like receptors, they inhibit the activity of adenylate cyclase through the Gαi protein, reduce the production of cyclic adenosine monophosphate (cAMP), and thus inhibit the PKA signaling pathway. The D_2_ receptor also has the function of regulating potassium and calcium ions and can directly regulate the excitability of neurons. The D_2_ receptor is widely present in regions such as the striatum and hypothalamus, while the D_3_ and D_4_ receptors are mainly distributed in the limbic system and prefrontal lobe (Channer et al., 2023; Neve et al., 2004).

Numerous studies have pointed out that dopamine receptors and PKC have broad application potential in mental diseases such as Parkinson’s disease, schizophrenia and Alzheimer’s disease (AD). This review focuses on the signal transduction between dopamine receptors and PKC (Alam and Sharma, 2019; Brown et al., 2023; Lin et al., 2023; Lindgren et al., 2010; Liu et al., 2023; Shanmukha et al., 2024).

## The influence of dopamine receptors on PKC activity

### D1-like receptors activate PKC via the Gαq protein

**FIGURE 1:**
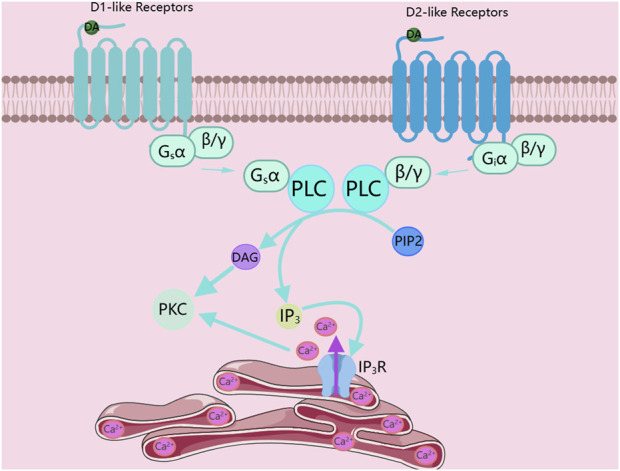
Dopamine receptors activate PKC. DA, Dopamine; PLC, phospholipase C; PIP2, phosphatidylinositol 4,5-bisphosphate; DAG, diacylglycerol; IP3, inositol 1,4,5-trisphosphate; IP3R, inositol 1,4,5-trisphosphate receptor; PKC, Protein kinase C. The figure was created with MedPeer (medpeer.cn).

Among G protein-coupled receptors, G proteins are the core of the entire signal transduction pathway. According to the different functions they mediate, they are classified into four types: Gs, Gi, G_12/13_, and Gq. The signal transduction of D1-like receptors mainly relies on coupling to the Gαs protein. The Gαs protein binds to the downstream adenylate cyclase, promoting the activity of adenylate cyclase. The activated adenylate cyclase catalyzes ATP in the cytoplasm into cAMP. As a second messenger, cAMP binds to downstream kinase proteins such as PKA, activating protein kinases like PKA, thus mediating the transduction of the signal pathway (Channer et al., 2023; Neve et al., 2004). In addition, D1-like receptors can also couple to the Gαq protein to drive the operation of the downstream signal pathway. When D1-like receptors couple to the Gαq protein instead of the Gαs protein, the Gαq protein binds to phospholipase C (PLC) and catalyzes the decomposition of phosphatidylinositol 4,5-bisphosphate (PIP2) into diacylglycerol and inositol 1,4,5-trisphosphate (IP3). IP3 binds to the inositol trisphosphate receptor (IP3R), which is a ubiquitous Ca^2+^-permeable channel that can mediate the release of Ca^2+^ from the endoplasmic reticulum. Then, DAG and Ca^2+^ jointly activate PKC, as shown in Figure 1 (Jackson et al., 2005; Paknejad and Hite, 2018).

### D2-like receptors activate PKC via the Gβγ subunit

Unlike D1-like receptors, the signal transduction of D2-like receptors mainly depends on coupling to the Gαi protein. The Gαi protein binds to the downstream adenylate cyclase to inhibit the activity of adenylate cyclase, thus preventing adenylate cyclase from catalyzing adenosine triphosphate (ATP) in the cytoplasm into cyclic AMP. Interestingly, the activation of PKC by D2-like receptors is mediated by the Gβγ subunit. In the D2-like receptor signal pathway, except for the different subtypes of G proteins, the pathways for activating PKC in the D1-like and D2-like receptor signal pathways are the same. The G_βγ_ subunit first binds to PLC and then catalyzes the decomposition of PIP2 into DAG and IP3. Since the IP3R is a ubiquitous Ca^2+^-permeable channel, after IP_3_ binds to IP_3_R, it can mediate the release of Ca^2+^ from the endoplasmic reticulum. Finally, DAG and Ca^2+^ jointly activate PKC, as shown in Figure 1 (Channer et al., 2023; Neve et al., 2004).

### Dopamine receptors inhibit PKC activity via β-arrestin

**FIGURE 2:**
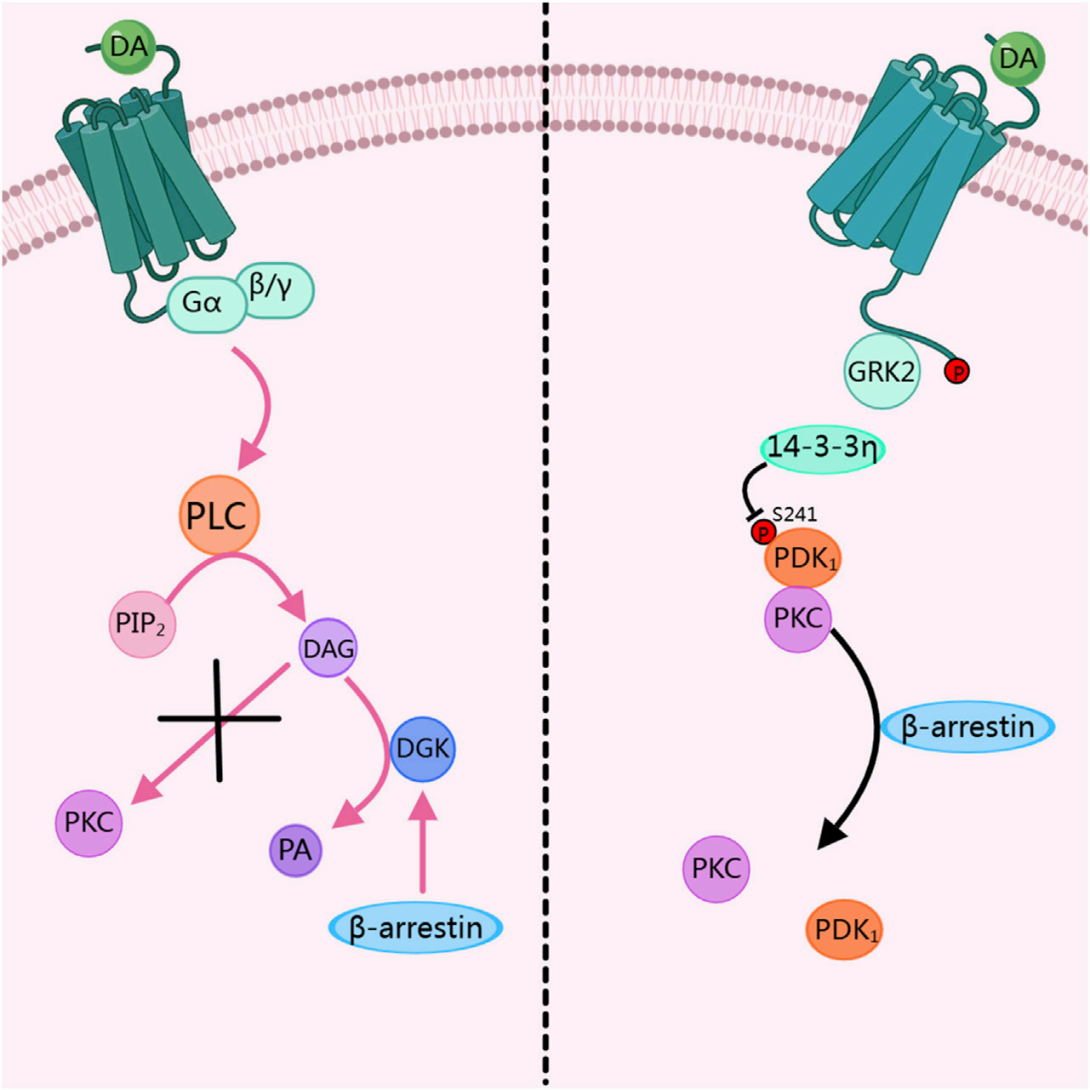
Dopamine receptors inhibit PKC activity. DA, Dopamine; PLC, phospholipase C; PIP2, phosphatidylinositol 4,5-bisphosphate; DAG, diacylglycerol; DGK, diacylglycerol kinase; PA, Phosphatidic acid; PKC, Protein kinase C; GRK2, G protein-coupled receptor kinase 2; PDK1, Pyruvate dehydrogenase kinase 1. The figure was created with MedPeer (medpeer.cn).

In the dopamine receptor signal pathway, when PKC is over-activated, it will affect physiological activities and even lead to diseases. Therefore, in a healthy system, the dopamine receptor pathway dynamically inhibits the activity of PKC to prevent diseases caused by over-activation of PKC. Research has shown that when PKC is activated by PMA, it binds to PDK 1 and undergoes membrane translocation. When dopamine stimulates D_2_R, β -arrestin 2 inhibits the binding of PDK1 to PKC, thereby suppressing the translocation and activation of PKC. This process involves GRK 2 and 14-3-3 η. Among them, the phosphorylation of the dopamine receptor by GRK 2 is a key step in inhibiting PKC activation. And 14-3-3 η dissociates PKC from Pyruvate dehydrogenase kinase 1 (PDK1) by inhibiting the phosphorylation of PDK 1-S241. This process belongs to heterologous regulation, as shown on the right side of Figure 2 (Zhang et al., 2020). Another study shows that β-arrestin activates diacylglycerol kinase (DGK), and then DGK mediates the conversion of DAG to phosphatidic acid (PA). Through this pathway, the activity of PKC is inhibited, thus suppressing the regulatory pathways mediated by PKC, this process belongs to homologous regulation, as shown on the left side of Figure 2 (Nelson et al., 2007).

## PKC regulates the release and reuptake of dopamine

Dopamine, as an endogenous agonist of dopamine receptors, activates dopamine receptors upon binding and initiates downstream signal transduction pathways. PKC indirectly regulates the downstream signal transduction of dopamine receptors by modulating the release and reuptake of dopamine (Sulzer et al., 2016).

### Dopamine release

In dopaminergic neurons, dopamine is stored in vesicles at the axon terminals. PKC promotes the fusion of synaptic vesicles with the cell membrane by phosphorylating synaptic proteins such as synapsin and syntaxin, thus enhancing dopamine release. The release of dopamine depends on the phosphorylation modification by PKC (Harsing et al., 2022). Some studies have demonstrated that PKC affects dopamine release by regulating calcium channels and related proteins of synaptic vesicles in presynaptic neurons (Luderman et al., 2015; Shoji-Kasai et al., 2002; Xue et al., 2009). Meanwhile, the release of dopamine relies on the structural change of membrane proteins, which allows Ca^2+^ influx, and the fusion of vesicles with nerve terminals or dendrites, releasing dopamine into the synaptic cleft through exocytosis. In this process, PKC phosphorylates calcium channel proteins, increasing the opening frequency of calcium channels and enhancing the influx of calcium ions, thereby promoting the release of dopamine vesicles (Herlitze et al., 2001). PKC not only regulates the release of vesicular dopamine but also participates in the regulation of non-vesicular dopamine release (Harsing et al., 2022). In addition, when treated with various PKC inhibitors (such as calphostin C, chelerythrine, and Ro31-8220), the amphetamine-induced dopamine release is inhibited, which conversely proves that PKC plays a crucial role in dopamine release (Gnegy, 2003).

### Dopamine reuptake

**FIGURE 3:**
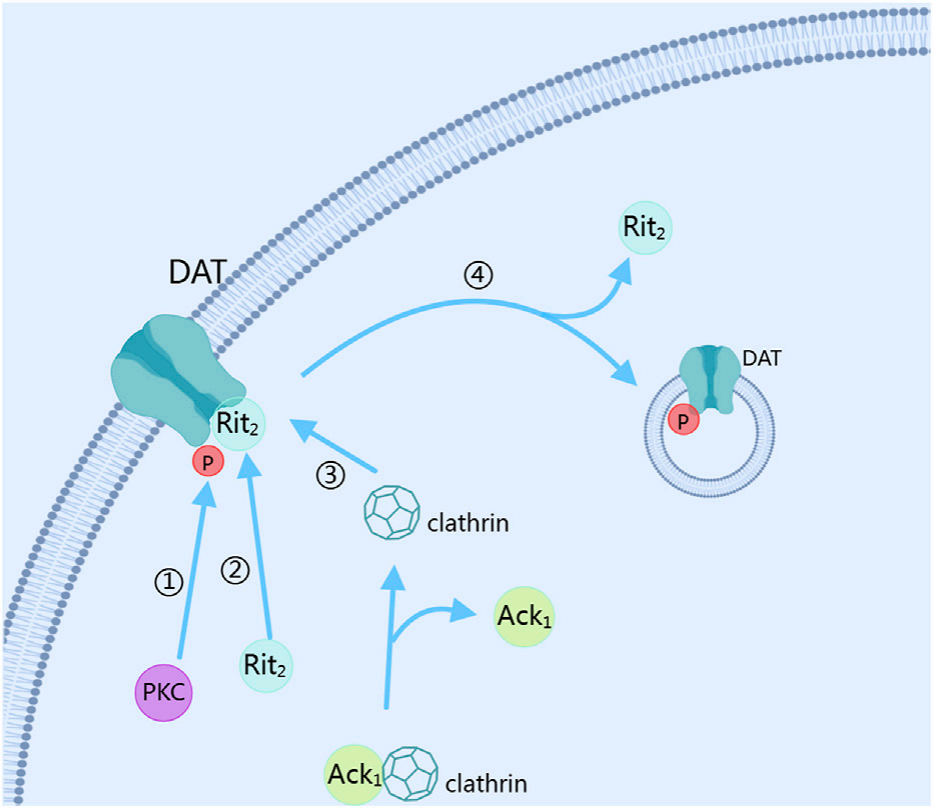
Dopamine reuptake. DAT, dopamine transporter; PKC, Protein kinase C; Ack1, activated Cdc42 (cell division cycle 42)-associated tyrosine kinase 1. The figure was created with MedPeer (medpeer.cn).

The reuptake of dopamine mainly depends on the dopamine transporter (DAT) (Mulvihill, 2019). After dopamine is released into the synaptic cleft through exocytosis, the dopamine transporter is activated to reduce the dopamine concentration in the synaptic cleft. PKC plays a key role in this process (Magee et al., 2021). PKC controls the expression level of DAT on the cell membrane by regulating its internalization and recycling, thus controlling the reuptake of dopamine (Foster and Vaughan, 2017; Julku et al., 2021). The reuptake function of DAT requires the co-regulation of phosphorylation and palmitoylation modifications (Julku et al., 2021). When the phosphorylation of DAT-Ser7 is low and the palmitoylation of DAT-C580 is high, the reuptake ability of DAT for dopamine is enhanced, and the PKC-mediated downregulation of DAT internalization is reduced. Conversely, when the phosphorylation of DAT-Ser7 is high and the palmitoylation of DAT-C580 is low, the reuptake ability of DAT for dopamine is weakened, and the PKC-mediated downregulation of DAT internalization is increased (Moritz et al., 2015). PKC can phosphorylate DAT, promoting its endocytosis and reducing the number of DAT expressed on the membrane surface, thereby decreasing the reuptake rate of dopamine and increasing the dopamine concentration in the synaptic cleft (Luis-Ravelo et al., 2021). In this process, when the amino acid residue at position 547 of DAT is mutated, the PKC-mediated phosphorylation of DAT is inhibited, indicating that the amino acid site at position 547 of DAT is the phosphorylation site of PKC (Quizon et al., 2016). Meanwhile, the PKC-mediated internalization of DAT depends on Ack1 and activated clathrin pits (Underhill and Amara, 2021). Interestingly, the activation of clathrin pits requires activated Cdc42 (cell division cycle 42)-associated tyrosine kinase 1 (Ack1), and PKC needs to inactivate Ack1 and dissociate it from clathrin before mediating the dissociation of DAT-Rit2 to accelerate DAT internalization (Fagan et al., 2020). In this process, the PKC-mediated internalization of DAT depends on its binding to Rit2, and the residues from position 587 to 596 of DAT are the sites that attract Rit2 binding (Fagan et al., 2020). At the same time, the residues from position 587 to 591 of DAT are the essential sites for PKC-induced accelerated internalization of DAT (Boudanova et al., 2008). By stimulating D3R with pramipexole, it was found that under short-term treatment, DAT translocates to the cell membrane under the mediation of PI3K and MAPK to reuptake dopamine in the synaptic cleft; while under long-term treatment, DAT on the cell membrane is phosphorylated and ubiquitinated, thus accelerating its internalization, reducing the expression of DAT on the cell membrane, and reducing the reuptake of dopamine by DAT. This process is PKCβ-dependent, as shown in Figure 3 (Luis-Ravelo et al., 2021). Conversely, when the activity of PKC is inhibited, the PKC-mediated internalization effect of DAT is also inhibited. PKC regulates the presynaptic dopamine release and phosphorylates the DAT to control the reuptake of dopamine, affecting the signal transmission and duration of dopamine, and ultimately influencing the downstream signal transduction of dopamine receptors.

## The relationship between PKC and downregulation of dopamine receptor

**FIGURE 4:**
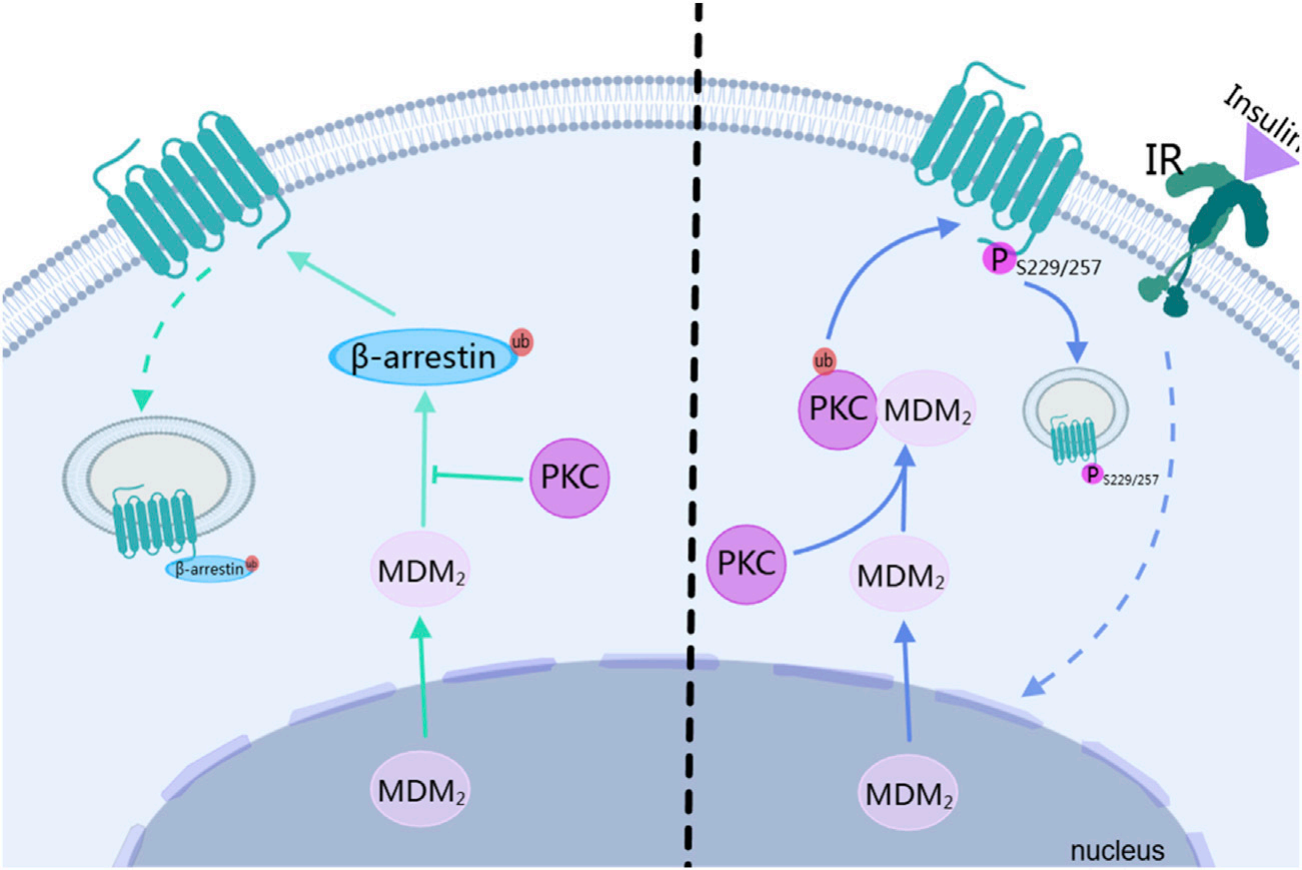
The relationship between PKC and downregulation of dopamine receptor. IR, insulin receptor; MDM2, Mouse doubleminute 2 homolog; ub, ubiquitin. The figure was created with MedPeer (medpeer.cn).

Endocytosis is not merely a simple process of taking up extracellular substances. It also occurs during the signal transduction of G protein-coupled receptors (GPCRs) (Zhang and Kim, 2017). In the endocytosis of GPCRs, it can be classified into two categories: one depends on the protein pathways such as GRK/arrestin, and the other requires second messenger-dependent kinases, like PKC(Kim, 2023). In the GRK/arrestin pathway, the β-arrestin2-mediated endocytosis of GPCRs depends on ubiquitination. Mouse doubleminute 2 homolog (Mdm2) is the ubiquitin ligase for β-arrestin2. After Mdm2 translocates from the nucleus to the cytoplasm and modifies β-arrestin2 through ubiquitination, β-arrestin2 translocates to the cell membrane to bind to GPCRs, initiating endocytosis. This process can be blocked by autophosphorylated PKCβⅡ. PKCβⅡ inhibits the binding of mdm2 to β-arrestin2, blocking the ubiquitination modification of β-arrestin2, thus inhibiting the endocytosis of GPCRs, as shown on the left side of Figure 4 (Zheng et al., 2015). In the protein kinase pathway such as that of PKC, the activated PKCβII phosphorylates D3R. Among them, the lysine residue at position 371 of PKCβII plays a crucial role in its kinase activity, which is the basis for its mediating the phosphorylation of D_3_R. The serine residues at positions 229 and 257 of D_3_R are potential phosphorylation sites of the receptor mediated by PKC. When these sites are mutated (such as S229/257A-D_3_R), D_3_R cannot undergo PKC-mediated phosphorylation, endocytosis, desensitization, and degradation. Receptor phosphorylation is a sufficient condition for endocytosis (Zhang et al., 2016). After the insulin receptor (IR) is activated during heterologous regulation, it promotes the ubiquitination of PKCβII mediated by Mdm2 through heterologous regulation. The activated PKCβII then phosphorylates D_3_R. Knocking down the clathrin heavy chain (CHC) inhibits the PKC-mediated endocytosis of D_3_R, indicating that clathrin is involved in this process, and the interaction between PKC and clathrin is necessary for the endocytosis of D_3_R, as shown on the right side of Figure 4 (Zeng et al., 2024).

The PKC-mediated endocytosis is also related to other proteins. For example, PKCβII interacts with actin, and this interaction affects its intracellular localization and function, which may further affect the endocytosis process of the receptor (Min et al., 2023a). The endocytic process of receptors is fundamentally dependent on GPCR phosphorylation. In homologous regulation, PKC modulates GPCR endocytosis by inhibiting receptor phosphorylation, whereas in heterologous regulation, PKC facilitates this process by promoting GPCR phosphorylation. Generally speaking, PKC modulates GPCR endocytosis by regulating their phosphorylation status.

## The relationship between PKC and dopaminergic diseases

### PKC and Parkinson’s disease

In Parkinson’s disease patients and various animal models, the activity of PKC in the nigrostriatal pathway shows significant abnormalities (Lin et al., 2023; Liu et al., 2023). In the early stage, the PKC activity may increase compensatorily. It attempts to resist the damaging factors such as oxidative stress and mitochondrial dysfunction that neurons are facing, and maintain the normal function of dopaminergic neurons by activating a series of intracellular anti-apoptotic signaling pathways. These include upregulating the expression of the anti-apoptotic protein B-cell lymphoma-2 (Bcl-2), inhibiting the activation of the pro-apoptotic protein Bax, and enhancing mitochondrial stability to reduce the production of reactive oxygen species (ROS) (Roshdy et al., 2024). However, as the disease progresses continuously, the long-term stress stimulation deteriorates the intracellular environment, and the activation pattern of PKC becomes disordered, with over-activation or abnormal translocation. Instead of playing its protective role, it may exacerbate the damage to neurons. For example, abnormally activated PKC may over-phosphorylate certain key proteins, such as tau protein, promoting its aggregation into neurofibrillary tangles, which destroys the cytoskeleton structure of neurons and disrupts axonal transport, thus affecting the normal metabolism and function maintenance of dopaminergic neurons. At the same time, changes in PKC activity will also feedback-regulate the function and expression of dopamine receptors, weakening the efficiency of dopamine signal transduction, further reducing the synthesis and release of dopamine, forming a vicious cycle and accelerating the disease progression (Kim and Kornberg, 2022).

### PKC and schizophrenia

PKC also plays an important role in the pathological process of schizophrenia. Research shows that the activity and expression levels of PKC in regions such as the cerebral cortex and hippocampus of schizophrenia patients are changed, and over-activation of PKC is relatively common (Arnsten, 2011; Pandey et al., 2020). This abnormal activation may stem from the continuous stimulation caused by hyperactivity of the dopamine system. Because the enhancement of dopaminergic signals can increase the production of DAG through the activation of a series of upstream signal molecules, such as PLC, and then over-activate PKC (Dean et al., 1997; Yabuki et al., 2019). The over-activated PKC will further exacerbate the disorder of the dopamine system. For example, PKC phosphorylates the D3 receptor, changing its coupling efficiency with the G protein, weakening the inhibitory effect of the D3 receptor on AC, causing an abnormal increase in intracellular cAMP levels, disrupting intracellular signal homeostasis, forming a vicious cycle, and continuously promoting the progression of the disease, affecting multiple functional dimensions such as the patient’s cognition, emotion, and behavior (Min et al., 2023b; Zeng et al., 2024).

### PKC and Alzheimer’s disease

In the AD model of 5XFAD mice, PKCη is specifically enriched in reactive astrocytes in the cortex and hippocampus, and regulates neuroinflammation through a negative feedback mechanism. Mechanistic studies have shown that the PKCη-mTOR-PP2A signaling axis inhibits the NF-κB-mediated inflammatory response through the following pathways: (1) mTORC2 mediates the phosphorylation and activation of PKCη at the T655 site; (2) The activated PKCη enhances the activity of Protein phosphatase 2 (PP2A); (3) PP2A inhibits the nuclear translocation of NF-κB-related proteins by dephosphorylating them, thereby downregulating the transcription of pro-inflammatory factors such as Interleukin-6 (IL-6). This regulatory pathway significantly alleviates the neuroinflammatory response in the AD model (Muraleedharan et al., 2021). Another study has shown that the protein level of PKCα in the brains of AD patients is significantly increased (by approximately 20%), and the phosphorylation level of its substrate synapse-associated protein 97 (SAP97) is increased by fourfold. PKCα remodels the brain phosphoproteome through enhanced catalytic activity (such as the M489V variant) or upregulated expression, leading to synaptic degeneration, a decrease in dendritic spines, and a decline in cognitive function. Its effect is independent of amyloid β-protein (Aβ) and synergizes with Aβ to accelerate the pathological process of AD. This indicates that the excessive activation of the PKCα signaling pathway may be a common feature of AD (Lordén et al., 2022). Meanwhile, some scholars have pointed out that PKC is activated by Aβ oligomers through an integrin β1-dependent pathway, and then phosphorylates the NR2B subunit, increasing its density and function in synapses, resulting in calcium signaling disorders and synaptic damage. This mechanism may manifest as a compensatory response in the early stages of AD, but with the progression of the disease, it ultimately exacerbates neuronal damage (Ortiz-Sanz et al., 2022). Bryostatin-1, as an agonist of PKCδ and PKCε, has entered clinical trials for the treatment of AD due to its favorable effects in animal models. Bryostatin-1 inhibits the accumulation of Aβ by activating PKC, thereby improving the symptoms of AD (Tian et al., 2023).

## Discussion

This review systematically and in-depthly explores the role of PKC in dopamine receptor signal transduction. It focuses on investigating the mechanism of PKC in dopamine receptor signal transduction. It is found that PKC is closely linked to the D1-like receptor signal pathway. It can not only respond to the activation of D1 receptors but also has a complex interaction with the D2-like receptor signal pathway to regulate the function of D2-like receptors. It further reveals the crucial role of PKC in related diseases such as Parkinson’s disease and schizophrenia. In Parkinson’s disease, the change in PKC activity is closely related to the degeneration and death of dopaminergic neurons. It is compensatorily activated in the early stage and becomes disordered in the later stage. It is involved in the regulation of anti-apoptotic signals and exacerbates neuron damage due to abnormal activation, providing a potential target for treatment. In schizophrenia, the hyperactivity of the dopamine system leads to the abnormal activation of PKC, forming a vicious cycle to promote the progression of the disease. Typical antipsychotic drugs indirectly regulate the activity of PKC to relieve symptoms, and the research and development of new drugs focus on the multi-target synergy of PKC, which is expected to break through the limitations of traditional drugs. Furthermore, studies have demonstrated reduced DAT uptake in patients with Parkinson’s disease and schizophrenia, with the degree of DAT reduction correlating positively with disease severity (Sampedro et al., 2021; Yang et al., 2024). In Alzheimer’s disease, the activities of different PKC subtypes play opposite roles. For example, the enhanced activity of PKCα exacerbates the symptoms of AD, while the enhanced activities of subtypes such as PKCη, PKCδ and PKCε reduce the accumulation of Aβ and improve the symptoms of AD. The role of PKC in AD is complex and subtype-specific. Future research needs to deeply analyze its molecular mechanism and develop precise treatment strategies targeting specific PKC subtypes.

## Future directions

Although current research has revealed the role of PKC in dopamine receptor signal transduction, many specific molecular mechanisms remain unexplored. For instance, in D1-like receptor signaling, it primarily couples with Gαs rather than Gαq. However, PKC activation requires the involvement of Gαq protein, and the Gαq family has not yet been thoroughly studied (Kamato et al., 2017). In the activation process of PKC, it is negatively regulated by β-arrestin, while in β-arrestin-mediated receptor internalization, PKC also exerts an influence. The molecular mechanisms underlying this mutual regulation remain unknown. Under pathological conditions, PKC dysfunction may contribute to diseases such as schizophrenia, Parkinson’s disease, and Alzheimer’s disease. However, research on PKC in dopaminergic disorders remains insufficient, and no novel drugs specifically targeting PKC have been developed.

In summary, future research could focus on the following directions: First, conducting detailed studies on Gαq proteins to elucidate their roles in cellular signaling and cell biology. Second, investigating the molecular mechanisms of the mutual regulation between PKC and β-arrestin, exploring their functions in cellular signal transduction and cell biology, and delving deeper into their interactions. Third, further exploring the role of the PKC protein family in the pathogenesis of dopaminergic disorders to provide a theoretical basis for targeted therapies. Finally, the PKC family holds significant research value as a drug target. The discovery and application of specific PKC activity biomarkers will greatly advance the development of therapeutic drugs based on PKC regulatory pathways. Additionally, establishing more optimized drug development strategies and strengthening systematic preclinical research evaluations are crucial steps to advance this field.

## References

[B1] Alam J. Sharma L. (2019). Potential enzymatic targets in Alzheimer's: a comprehensive review. Curr. Drug Targets 20, 316–339. 10.2174/1389450119666180820104723 30124150

[B2] Arnsten A. F. (2011). Prefrontal cortical network connections: Key site of vulnerability in stress and schizophrenia. Int. J. Dev. Neurosci. 29, 215–223. 10.1016/j.ijdevneu.2011.02.006 21345366 PMC3115784

[B3] Boudanova E. Navaroli D. M. Stevens Z. Melikian H. E. (2008). Dopamine transporter endocytic determinants: Carboxy terminal residues critical for basal and PKC-stimulated internalization. Mol. Cell. Neurosci. 39, 211–217. 10.1016/j.mcn.2008.06.011 18638559 PMC2585501

[B4] Brown J. Grayson B. Neill J. C. Harte M. Wall M. J. Ngomba R. T. (2023). Oscillatory deficits in the sub-chronic PCP rat model for schizophrenia are reversed by mGlu5 receptor-positive allosteric modulators VU0409551 and VU0360172. Cells 12, 919. 10.3390/cells12060919 36980260 PMC10047164

[B5] Channer B. Matt S. M. Nickoloff-Bybel E. A. Pappa V. Agarwal Y. Wickman J. (2023). Dopamine, immunity, and disease. Pharmacol. Rev. 75, 62–158. 10.1124/pharmrev.122.000618 36757901 PMC9832385

[B6] Dean B. Opeskin K. Pavey G. Hill C. Keks N. (1997). Changes in protein kinase C and adenylate cyclase in the temporal lobe from subjects with schizophrenia. J. Neural Transm. 104, 1371–1381. 10.1007/bf01294738 9503283

[B7] Fagan R. R. Kearney P. J. Sweeney C. G. Luethi D. Schoot Uiterkamp F. E. Schicker K. (2020). Dopamine transporter trafficking and Rit2 GTPase: mechanism of action and *in vivo* impact. J. Biol. Chem. 295, 5229–5244. 10.1074/jbc.ra120.012628 32132171 PMC7170531

[B8] Foster J. D. Vaughan R. A. (2017). Phosphorylation mechanisms in dopamine transporter regulation. J. Chem. Neuroanat. 83-84, 10–18. 10.1016/j.jchemneu.2016.10.004 27836487 PMC6705611

[B9] Freeley M. Kelleher D. Long A. (2011). Regulation of Protein Kinase C function by phosphorylation on conserved and non-conserved sites. Cell. Signal. 23, 753–762. 10.1016/j.cellsig.2010.10.013 20946954

[B10] Gnegy M. E. (2003). The effect of phosphorylation on amphetamine-mediated outward transport. Eur. J. Pharmacol. 479, 83–91. 10.1016/j.ejphar.2003.08.059 14612140

[B11] Gruber T. Hermann-Kleiter N. Pfeifhofer-Obermair C. Lutz-Nicoladoni C. Thuille N. Letschka T. (2009). PKC theta cooperates with PKC alpha in alloimmune responses of T cells *in vivo* . Mol. Immunol. 46, 2071–2079. 10.1016/j.molimm.2009.02.030 19356803

[B12] Halet G. (2004). PKC signaling at fertilization in mammalian eggs. Biochimica Biophysica Acta (BBA) - Mol. Cell Res. 1742, 185–189. 10.1016/j.bbamcr.2004.09.012 15590069

[B13] Harsing L. G. Knoll J. Miklya I. (2022). Enhancer regulation of dopaminergic neurochemical transmission in the striatum. Int. J. Mol. Sci. 23, 8543. 10.3390/ijms23158543 35955676 PMC9369307

[B14] He S. Li Q. Huang Q. Cheng J. (2022). Targeting protein kinase C for cancer therapy. Cancers (Basel) 14, 1104. 10.3390/cancers14051104 35267413 PMC8909172

[B15] Herlitze S. Zhong H. Scheuer T. Catterall W. A. (2001). Allosteric modulation of Ca ^2+^ channels by G proteins, voltage-dependent facilitation, protein kinase C, and Ca _v_ β subunits. Proc. Natl. Acad. Sci. U. S. A. 98, 4699–4704. 10.1073/pnas.051628998 11296298 PMC31897

[B16] Jackson A. Sedaghat K. Minerds K. James C. Tiberi M. (2005). Opposing effects of phorbol-12-myristate-13-acetate, an activator of protein kinase C, on the signaling of structurally related human dopamine D1 and D5 receptors. J. Neurochem. 95, 1387–1400. 10.1111/j.1471-4159.2005.03476.x 16313517

[B17] Julku U. H. Jäntti M. Svarcbahs R. Myöhänen T. T. (2021). Prolyl oligopeptidase regulates dopamine transporter oligomerization and phosphorylation in a PKC- and ERK-independent manner. Int. J. Mol. Sci. 22, 1777. 10.3390/ijms22041777 33579026 PMC7916783

[B18] Kamato D. Mitra P. Davis F. Osman N. Chaplin R. Cabot P. J. (2017). Ga(q) proteins: Molecular pharmacology and therapeutic potential. Cell Mol. Life Sci. 74, 1379–1390. 10.1007/s00018-016-2405-9 27815595 PMC11107756

[B19] Kawahata I. Fukunaga K. (2023). Endocytosis of dopamine receptor: signaling in brain. Prog. Mol. Biol. Transl. Sci. 196, 99–111. 10.1016/bs.pmbts.2022.09.005 36813367

[B20] Kim H. R. Gallant C. Morgan K. G. (2013). Regulation of PKC autophosphorylation by calponin in contractile vascular smooth muscle tissue. BioMed Res. Int. 2013, 1–9. 10.1155/2013/358643 PMC3852320 24350264

[B21] Kim K. M. (2023). Unveiling the differences in signaling and regulatory mechanisms between dopamine D(2) and D(3) receptors and their impact on behavioral sensitization. Int. J. Mol. Sci. 24, 6742. 10.3390/ijms24076742 37047716 PMC10095578

[B22] Kim P. M. Kornberg M. D. (2022). Targeting PKC in microglia to promote remyelination and repair in the CNS. Curr. Opin. Pharmacol. 62, 103–108. 10.1016/j.coph.2021.11.008 34965482

[B23] Lin X. M. Pan M. H. Sun J. Wang M. Huang Z. H. Wang G. (2023). Membrane phospholipid peroxidation promotes loss of dopaminergic neurons in psychological stress-induced Parkinson's disease susceptibility. Aging Cell 22, e13970. 10.1111/acel.13970 37622525 PMC10577563

[B24] Lindgren H. S. Andersson D. R. Lagerkvist S. Nissbrandt H. Cenci M. A. (2010). L-DOPA-induced dopamine efflux in the striatum and the substantia nigra in a rat model of Parkinson's disease: Temporal and quantitative relationship to the expression of dyskinesia. J. Neurochem. 112, 1465–1476. 10.1111/j.1471-4159.2009.06556.x 20050978

[B25] Liu L. L. Han Y. Zhang Z. J. Wang Y. Q. Hu Y. W. Kaznacheyeva E. (2023). Loss of DJ-1 function contributes to Parkinson's disease pathogenesis in mice via RACK1-mediated PKC activation and MAO-B upregulation. Acta Pharmacol. Sin. 44, 1948–1961. 10.1038/s41401-023-01104-8 37225849 PMC10545772

[B26] Lordén G. Wozniak J. M. Doré K. Dozier L. E. Cates-Gatto C. Patrick G. N. (2022). Enhanced activity of Alzheimer disease-associated variant of protein kinase Cα drives cognitive decline in a mouse model. Nat. Commun. 13, 7200. 10.1038/s41467-022-34679-7 36418293 PMC9684486

[B27] Luderman K. D. Chen R. Ferris M. J. Jones S. R. Gnegy M. E. (2015). Protein kinase C beta regulates the D_2_-like dopamine autoreceptor. Neuropharmacology 89, 335–341. 10.1016/j.neuropharm.2014.10.012 25446677 PMC4293343

[B28] Luis-Ravelo D. Fumagallo-Reading F. Castro-Hernandez J. Barroso-Chinea P. Afonso-Oramas D. Febles-Casquero A. (2021). Prolonged dopamine D(3) receptor stimulation promotes dopamine transporter ubiquitination and degradation through a PKC-dependent mechanism. Pharmacol. Res. 165, 105434. 10.1016/j.phrs.2021.105434 33484816

[B29] Magee C. P. Le B. D. Siripathane Y. H. Wilkins D. G. Hanson G. R. Fleckenstein A. E. (2021). Methcathinone decreases dopamine transporter function: role of protein kinase C. J. Neurochem. 159, 116–127. 10.1111/jnc.15483 34320222

[B30] Mellor H. Parker P. J. (1998). The extended protein kinase C superfamily. Biochem. J. 332 (Pt 2), 281–292. 10.1042/bj3320281 9601053 PMC1219479

[B31] Min X. Wang S. Zhang X. Sun N. Kim K. M. (2023a). PKCβII activation requires nuclear trafficking for phosphorylation and Mdm2-mediated ubiquitination. Life Sci. Alliance 6, e202201748. 10.26508/lsa.202201748 36717249 PMC9887771

[B32] Min X. Zhang X. Wang S. Kim K. M. (2023b). Activation of PKCβII through nuclear trafficking guided by βγ subunits of trimeric G protein and 14-3-3ε. Life Sci. 312, 121245. 10.1016/j.lfs.2022.121245 36503900

[B33] Mishra A. Singh S. Shukla S. (2018). Physiological and functional basis of dopamine receptors and their role in neurogenesis: possible implication for Parkinson's disease. J. Exp. Neurosci. 12, 1179069518779829. 10.1177/1179069518779829 29899667 PMC5985548

[B34] Moritz A. E. Rastedt D. E. Stanislowski D. J. Shetty M. Smith M. A. Vaughan R. A. (2015). Reciprocal phosphorylation and palmitoylation control dopamine transporter kinetics. J. Biol. Chem. 290, 29095–29105. 10.1074/jbc.m115.667055 26424792 PMC4661421

[B35] Mulvihill K. G. (2019). Presynaptic regulation of dopamine release: role of the DAT and VMAT2 transporters. Neurochem. Int. 122, 94–105. 10.1016/j.neuint.2018.11.004 30465801

[B36] Muraleedharan A. Rotem‐Dai N. Strominger I. Anto N. P. Isakov N. Monsonego A. (2021). Protein kinase C eta is activated in reactive astrocytes of an Alzheimer's disease mouse model: evidence for its immunoregulatory function in primary astrocytes. Glia 69, 697–714. 10.1002/glia.23921 33068318

[B37] Nelson C. D. Perry S. J. Regier D. S. Prescott S. M. Topham M. K. Lefkowitz R. J. (2007). Targeting of diacylglycerol degradation to M1 muscarinic receptors by ß-arrestins. Science 315, 663–666. 10.1126/science.1134562 17272726

[B38] Neve K. A. Seamans J. K. Trantham-Davidson H. (2004). Dopamine receptor signaling. J. Recept. Signal Transduct. 24, 165–205. 10.1081/rrs-200029981 15521361

[B39] Newton A. C. (2001). Protein kinase C: Structural and spatial regulation by phosphorylation, cofactors, and macromolecular interactions. Chem. Rev. 101, 2353–2364. 10.1021/cr0002801 11749377

[B40] Ortiz-Sanz C. Balantzategi U. Quintela-López T. Ruiz A. Luchena C. Zuazo-Ibarra J. (2022). Amyloid β/PKC-dependent alterations in NMDA receptor composition are detected in early stages of Alzheimer´s disease. Cell Death Dis. 13, 253. 10.1038/s41419-022-04687-y 35306512 PMC8934345

[B41] Paknejad N. Hite R. K. (2018). Structural basis for the regulation of inositol trisphosphate receptors by Ca(2+) and IP(3). Nat. Struct. Mol. Biol. 25, 660–668. 10.1038/s41594-018-0089-6 30013099 PMC6082148

[B42] Pandey G. N. Rizavi H. S. Ren X. (2020). Protein and mRNA expression of protein kinase C (PKC) in the postmortem brain of bipolar and schizophrenic subjects. J. Psychiatric Res. 130, 362–371. 10.1016/j.jpsychires.2020.07.019 PMC7554203 32882578

[B43] Quizon P. M. Sun W. L. Yuan Y. Midde N. M. Zhan C. G. Zhu J. (2016). Molecular mechanism: The human dopamine transporter histidine 547 regulates basal and HIV-1 tat protein-inhibited dopamine transport. Sci. Rep. 6, 39048. 10.1038/srep39048 27966610 PMC5155291

[B44] Roshdy M. Zaky D. A. Abbas S. S. Abdallah D. M. (2024). Niacin, an innovative protein kinase-C-dependent endoplasmic reticulum stress reticence in murine Parkinson's disease. Life Sci. 351, 122865. 10.1016/j.lfs.2024.122865 38914304

[B45] Saito N. Shirai Y. (2002). Protein kinase C (PKC): function of neuron specific isotype. J. Biochem. 132, 683–687. 10.1093/oxfordjournals.jbchem.a003274 12417016

[B46] Sajan M. P. Hansen B. C. Acevedo-Duncan M. Kindy M. S. Cooper D. R. Farese R. V. (2021). Roles of hepatic atypical protein kinase C hyperactivity and hyperinsulinemia in insulin-resistant forms of obesity and type 2 diabetes mellitus. MedComm 2, 3–16. 10.1002/mco2.54 34766133 PMC8491214

[B47] Sampedro F. Marín-Lahoz J. Martínez-Horta S. Camacho V. Lopez-Mora D. A. Pagonabarraga J. (2021). Extrastriatal SPECT-DAT uptake correlates with clinical and biological features of *de novo* Parkinson's disease. Neurobiol. Aging 97, 120–128. 10.1016/j.neurobiolaging.2020.10.016 33212336

[B48] Shanmukha S. Godfrey W. H. Gharibani P. Lee J. J. Guo Y. Deng X. (2024). TPPB modulates PKC activity to attenuate neuroinflammation and ameliorate experimental multiple sclerosis. Front. Cell Neurosci. 18, 1373557. 10.3389/fncel.2024.1373557 38841204 PMC11150779

[B49] Shoji‐Kasai Y. Itakura M. Kataoka M. Yamamori S. Takahashi M. (2002). Protein kinase C-mediated translocation of secretory vesicles to plasma membrane and enhancement of neurotransmitter release from PC12 cells. Eur. J. Neurosci. 15, 1390–1394. 10.1046/j.1460-9568.2002.01972.x 11994133

[B50] Su T. Straight S. Bao L. Xie X. Lehner C. L. Cavey G. S. (2013). PKC ε phosphorylates and mediates the cell membrane localization of RhoA. ISRN Oncol. 2013, 1–9. 10.1155/2013/329063 PMC3804392 24191200

[B51] Sulzer D. Cragg S. J. Rice M. E. (2016). Striatal dopamine neurotransmission: Regulation of release and uptake. Basal Ganglia 6, 123–148. 10.1016/j.baga.2016.02.001 27141430 PMC4850498

[B52] Tian Z. Lu X. T. Jiang X. Tian J. (2023). Bryostatin-1: A promising compound for neurological disorders. Front. Pharmacol. 14, 1187411. 10.3389/fphar.2023.1187411 37351510 PMC10282138

[B53] Underhill S. M. Amara S. G. (2021). Acetylcholine receptor stimulation activates protein kinase C mediated internalization of the dopamine transporter. Front. Cell Neurosci. 15, 662216. 10.3389/fncel.2021.662216 33897375 PMC8062973

[B54] Xue R. Zhao Y. Chen P. (2009). Involvement of PKC alpha in PMA-induced facilitation of exocytosis and vesicle fusion in PC12 cells. Biochem. Biophysical Res. Commun. 380, 371–376. 10.1016/j.bbrc.2009.01.105 19250646

[B55] Yabuki Y. Wu L. Fukunaga K. (2019). Cognitive enhancer ST101 improves schizophrenia-like behaviors in neonatal ventral hippocampus-lesioned rats in association with improved CaMKII/PKC pathway. J. Pharmacol. Sci. 140, 263–272. 10.1016/j.jphs.2019.07.015 31474557

[B56] Yang K. C. Yang B. H. Liu M. N. Liou Y. J. Chou Y. H. (2024). Cognitive impairment in schizophrenia is associated with prefrontal-striatal functional hypoconnectivity and striatal dopaminergic abnormalities. J. Psychopharmacol. 38, 515–525. 10.1177/02698811241257877 38853592

[B57] Yang Y. (2021). Functional selectivity of dopamine D(1) receptor signaling: retrospect and prospect. Int. J. Mol. Sci. 22, 11914. 10.3390/ijms222111914 34769344 PMC8584964

[B58] Zeng X. Wu C. Cao Y. Li H. Zhang X. (2024). Mdm2-mediated ubiquitination of PKCβII is responsible for insulin-induced heterologous desensitization of dopamine D(3) receptor. FEBS Lett. 598, 400–414. 10.1002/1873-3468.14815 38302840

[B59] Zhang X. Kim K. M. (2017). Multifactorial regulation of G protein-coupled receptor endocytosis. Biomol. and Ther. 25, 26–43. 10.4062/biomolther.2016.186 PMC5207461 28035080

[B60] Zhang X. Min X. Zhu A. Kim K. M. (2020). A novel molecular mechanism involved in the crosstalks between homologous and PKC-mediated heterologous regulatory pathway of dopamine D(2) receptor. Biochem. Pharmacol. 174, 113791. 10.1016/j.bcp.2020.113791 31917245

[B61] Zhang X. Sun N. Zheng M. Kim K. M. (2016). Clathrin-mediated endocytosis is responsible for the lysosomal degradation of dopamine D3 receptor. Biochem. Biophysical Res. Commun. 476, 245–251. 10.1016/j.bbrc.2016.05.104 27240955

[B62] Zheng M. Zhang X. Guo S. Zhang X. Choi H. J. Lee M. Y. (2015). PKCβII inhibits the ubiquitination of β-arrestin2 in an autophosphorylation-dependent manner. FEBS Lett. 589, 3929–3937. 10.1016/j.febslet.2015.10.031 26545496

